# Foreign

**Published:** 1841-07-24

**Authors:** 


### FOREIGN.

*Case of Dislocation of the Cervical Vertebræ cured.* By Dr. Schuk, of Vienna.—A man, 24 years of age, whilst engaged at his work on the 5th of December, 1838, twisted his head suddenly round, in consequence of one of his companions roaring into his ear, when he instantly felt something give way in his neck, and found it impossible to move his head. Next morning, when he applied for assistance, his face was swollen, his head turned to the right, and bent down towards the shoulder. His neck was slightly arched on the left side, but hollowed on the right. He complained of pain, which was augmented on pressure over the seat of the third, fourth, and fifth cervical vertebræ, and he was unable to move the head in any direction; every attempt to do so gave pain. The direction of the spinous processes of these vertebræ could not be accurately ascertained. He complained of weakness of the right arm, and could only raise it with great effort. The other functions of the body appeared to be natural. It was thus apparent that partial dislocation of some of the cervical vertebræ existed, and some attempts were made to reduce it by drawing the head directly upwards, the trunk of the body being held fixed; these, however, failed, but they gave no pain.

On the 7th of December, the weakness and numbness of the right arm were greater. Now efforts were therefore made to effect the reduction of the dislocation. The patient was laid in the horizontal posture, the shoulders were held fixed by means of folded sheets, whilst a towel was passed under the chin, in order to allow of a greater force being used for the extension; an assistant supported the occiput with both his hands. Extension was then made and gradually augmented, till the patient and assistant felt a snap as of two bones meeting. The extension was then gradually relaxed, when it was found that the head was restored to its normal position, and the power of moving it was restored. The weakness of the limb, however, remained, and was even worse next day. On the 9th he complained of vertigo and starting during his sleep, and his pulse was quick. For this he was bled to a considerable extent, which induced fainting and convulsions. He passed, however, a good night, and next day his pulse was nearly natural, the vertigo was gone, and he had partially regained the use of the right arm. He left the hospital cured on the 13th.—*Ed. Med. and Surg. Journ.*, from *Medizinische Jahrbuch des Osterreichischen Staates*, vol. xxx, 1840.

---

*On the best mode of treating cases of Poisoning by Arsenic.* By M. Orfila.—Rasori and Giacomini sometime ago advanced, that the ordinary antiphlogistic treatment usually practised in cases of poisoning by arsenic was decidedly hurtful, and that, from their experiments on forty-seven dogs, a stimulant and tonic plan of treatment was that best fitted to effect a cure. With the view of ascertaining the truth of this statement, M. Orfila, in the presence of a commission of the Royal Academy of Medicine, and of many of his friends, experimented on one hundred and fifty-seven dogs; and the following is a short abstract of his extended inquiry:

1. Twelve dogs had their œsophagus tied, and the ligature left on for thirty hours. When removed at the end of this period, the animals eat and drank freely, and seemed no ways injured; and the wound in the neck healed in a few days. The experiment was for the purpose of ascertaining what amount of suffering was to be attributed to the ligature in the subsequent experiments, where the œsophagus was tied to prevent the poison being vomited.

2. To three dogs were administered the stimulant and tonic mixtures, said by the Italian physicians to be so successful in the treatment of arsenical poisoning; but they were found in every case to produce death in a few hours, the animals exhibiting all the symptoms of intoxication, and also occasionally acute internal pains. The mixture was composed of eight ounces of beef tea, two ounces of brandy, and the same quantity of wine.

3. Arsenic in powder was introduced into the subcutaneous cellular tissue of the inside of the thighs of thirty-four dogs, and the following was the result:—Two grains was the quantity used for each dog; and the first five being left without any treatment, died in from thirty to forty hours. Ten dogs were treated by the Italian tonic plan and all died. Four were allowed lukewarm water, but also died, having passed very little urine. Seven dogs were bled about six or seven hours after the introduction of the poison; but they all died. Six dogs had diuretic medicines administered to them, composed of six pounds of water, one ponnd of white wine, and one ounce of saltpetre, with the occasional addition of a little Seltzer water, and all recovered. They passed large quantities of urine, which contained arsenic, as was ascertained by analysing it in the apparatus of Marsh. Four other dogs, treated by the saltpetre water died; but they scarcely passed any urine.

4. Forty-one dogs were made to swallow arsenic in powder, in quantities varying from three and a half to ten and a half grains, with the following results:—Four dogs whose œsophagus was not tied recovered without any treatment, having expelled the poison by free vomiting. Four dogs whose œsophagus was tied died in a longer or shorter time. Four dogs who were allowed to vomit recovered, although subjected to the stimulant treatment. A ligature was kept round the œsophagus of seven dogs for a variable length of time, from two to thirty hours, and the animals were subjected to the Italian stimulant treatment; four only died. Of those which recovered, two vomited after the removal of the ligature, which in one was after two hours and three-quarters, and in the others after five hours and a half, but all passed an abundant quantity of urine. Four dogs had quinine administered to them in a decoction of cinchona bark; but their œsophagus was tied, and they all died. No urine, or only very little, was passed by them. Nine dogs who were subjected to the aqueous treatment, and were allowed to vomit, all recovered. They all vomited and passed urine freely. Of nine dogs who were bled, seven recovered. In four of them the œsophagus was tied for three, four, and five hours, and blood-letting was the only remedy used. The other three were allowed to vomit.

5. In this series of experiments, the arsenic was dissolved in water, and then introduced into the stomach, the same quantities being used as in the former series of experiments. Seven dogs, three of whom had the œsophagus tied for three hours, died in a few hours, though several of them vomited. Eighteen dogs, who had each about four grains of arsenic administered to them, and had the œsophagus tied for from forty minutes to two hours, all died, in spite of the Italian stimulant treatment. One large dog vomited a part of the solution, and recovered; it had also been subjected to the stimulant treatment. Eight dogs, which vomited freely a few minutes after taking the poison, and were subjected to the aqueous treatment, recovered. They all passed large quantities of urine. Another dog, which did not vomit for one hour, died, though it was similarly treated. Two others, which had the œsophagus tied, the one for three-quarters of an hour, the other for fifty minutes, died. Thirteen dogs, were bled, but only two recovered. Nine dogs were bled, and had also hot water given to them, and seven recovered. The two which died had the œsophagus tied, and vomiting, of course, prevented for fifty minutes.

6. This series of experiments was undertaken with the view of ascertaining whether the exciting a copious diuresis would have the effect of obviating a fatal result, when other poisonous agents were administered. Tartrate of antimony was the first poison selected: and M. Orfila found, that one grain and a half introduced into the cellular substance of the thigh produced death in four dogs in the space of from seventeen to thirty-six hours. Five dogs were similarly poisoned, but had diuretic drinks administered to them, and four recovered. They passed large quantities of urine, which was ascertained to contain antimony. The dog which died had passed no urine.

7. Opium was the poison next experimented on; and from fifteen to ninety grains of its watery extract were administerd to twelve dogs, in some of the cases being applied to the cellular tissue of the thigh, at other times introduced into the stomach. But these dogs, though subjected to the diuretic treatment, passed no urine, so that he was unable to ascertain whether this poison, like the metallic salts, could be removed from the system by the action of the kidneys of these animals. He has, however, been able to ascertain that opium is passed off with the urine, having several times detected morphia in that fluid; and he thinks, however, that in man a diuretic plan of treatment might with propriety be adopted, as one means of freeing the system from the poison, diuresis being more easily induced in him, when labouring under the effects of opium, than it is in the dog.

The conclusions which M. Orfila draws from the above experiments are so very obvious, that it is unnecessary to detail them here. In poisoning with arsenic or other metallic salts, after free vomiting, the great object is to aid the expulsion of the poison from the system by exciting full and free diuresis. The Italian stimulant plan appears to be worse than useless. —*Ibid., from Bulletin de l'Academie Royale de Medecine.*

*Case of Poisoning with the Acetate of Lead, in which the poison was detected in the Urine.* By MM. Orfila and Villeneuve.—A girl, in a fit of despair, swallowed between eight and ten drachms of the superacetate of lead. She was speedily affected with prostration of strength, paleness and coldness of the surface of the body, and faintings, which symptoms were in a short time succeeded by vomiting and precordial anxiety. Sulphate of soda was given in large and repeated doses, and was followed by copious alvine evacuations. Under this treatment the poisonous symptoms went off, and the temperature of the body returned. The urine which the girl passed twenty-five hours after swallowing the poisonous dose was examined by M. Orfila, who extracted from it a considerable quantity of lead, showing that the poison which had been absorbed was thrown off from the system by means of the kidneys.

M. Lassaigne, of Alfort, made a number of experiments at the veterinary school there, with the view of ascertaining in what secretions or organs the lead with which animals have been poisoned is found. He constantly met with it in large quantity in the venous blood and in the urine of living animals, and in the liver and kidneys after death.

MM. Chevallier and Brichateau have examined the urine of the workmen in the lead manufactories, especially at the time when they were affected by this metal, but have as yet been unable to detect it even in the most minute quantity in that fluid. M. Orfila, also, was unable to detect its presence in the body of a child supposed to have been poisoned from breathing its vapours.—*Ibid, from Bulletin de l'Academie Royale de Medecine.*

---

*Case of Poisoning by Tobacco.*—By M. Tavignot.—A strong man, of 55 years of age, had a tobacco enema administered to him for the relief of ascarides in the rectum. The enema was ordered to be composed of one drachm and a half (60 centigrammes) of tobacco leaves in about six ounces of water, but by mistake 15 drachms (60 grammes) of tobacco were used, and administered before the mistake was discovered. Seven or eight minutes had scarcely elapsed from the period of its administration before stupor, headach, and extreme paleness of the face made their appearance; pain was complained of in the abdomen, the speech became thick and indistinct, and slight convulsive movements were observed. A purgative enema was immediately administered, stimuli and strong coffee were also given, cloths dipped in cold water were applied to the head, and sinapisms to various parts of the body. He was also bled pretty freely.

Notwithstanding all these means, the paleness of the face and surface of the body increased, the expression of the face was indicative of pain and stupor, the pupils of the eyes were natural, the respiration became more and more laborious and slow; his intellectual faculties seemed to be greatly weakened, but he still occasionally understood questions which were put to him, though unable to answer to them distinctly; convulsive tremors were first observed in the arms, but soon extended to the legs and trunk of the body, and went on increasing in severity for six or seven minutes, after which a state of complete prostration came on, attended with slow very painful respiration. Well marked coma, with complete relaxation of all the muscles of the body, preceded the fatal termination, which took place about eighteen minutes after the administration of the enema. The pulse stood at 68 before the blood-letting, and 44 after it. No vomiting occurred in this case.—*Ibid, from Gazette Medicale de Paris.*

---

*On the length and strength of the Umbilical Cord at the full term of Pregnancy.* By Dr. Negrier. (*Annalesd' Hygiene Publique*, January, 1841.)—A girl of bad character was accused of having strangled her child by means of the umbilical cord, before it was completely expelled from the uterus. As there was a difference of opinion amongst the medical men as to the possibiliiy of the umbilicated cord possessing sufficient strength or length for this purpose, Dr. Negrier performed a number of experiments, for the purpose of ascertaining the strength of the cord, and measured it in 166 cases to arrive at its average length.

Of the 166 cases it was remarked that in 144 the umbilical cord floated free within the uterus; in twenty cases it was rolled around the neck of the child; in one it was round the shoulders; and in one between the thighs, the breech presenting in this case. Ninety-eight of the umbilical cords were not varicose, and sixty-eight were varicose. As to length, twenty-eight were 17 inches long, 112 were from 17 to 25½ inches long, and twenty-six above that length.

The resistance of the umbilical cords was ascertained by attaching weights to one end of the cord until it ruptured, the weights being always attached to the placentar extremity. About one-half of the cords were passed by their middle over a round bar, and weights attached until they gave way; the other half of the number were rolled once and a-half round the same bar, covered with linen, so as to bring it to the diameter of the child's neck, when it was found that these supported a greater weight than those over the plain bar. The varicose umbilical cords were ruptured with a lesser weight than the sound cords, and generally gave way at one of the varicose dilations. The mean weight which these varicose cords supported before they gave way was 8 pounds Troy; the most restraint supported 14 pounds 17 ounces. The medium resistance of the non-varicose umbilical cords was 14 pounds 4 ounces Troy; but one cord required 25 pounds 3 ounces to rupture it.

Dr. Negrier next made a few experiments to ascertain what weights suspended round the neck of an adult would produce such a degree of compression as to cause unpleasant feelings or strangulation. A weight of 8 pounds was suspended to a cord passed once and a-half round the neck, the back of the neck being upwards. The respiration was rendered difficult, and the brain strongly congested in two minutes. Vertigo commenced soon afterwards. The respiration, however, could be continued with difficulty. When the face was placed upwards the effects of the congestion were more rapid; the respiration was much impeded, but was still possible; but Dr. Negrier thought that death would have resulted if this position was maintained for a quarter of an hour.

When the experiment was made with a weight of 13 pounds, and the face downwards, rapid congestion of all the vessels of the head took place; the eyes became injected, and filled with tears; the respiration was very laborious, but was still possible. It was, however, dangerous to continue the experiment for two minutes.

When the same experiment was repeated, but with the face looking upwards, the strangulation was almost complete. Respiration was so impeded that Dr. Negrier thinks death would have resulted in less five minutes.

From these facts, he infers that the umbilical cord is both long enough and strong enough to produce strangulation in a new-born infant, by being twisted round its neck after the head is delivered. A force applied to a cord equal to 12 pounds would strangle an adult in five minutes, and a much lesser force would strangle a child.—*Ibid.*

---

*Case of severe epilepsy arising from enlargement of the nerves.*—M. D., æt 21, was brought to the hospital on the evening of the 17th of Feb. 1840, with the left hand lacerated by machinery. The first three fingers, and half of the little finger, were carried away; and the inner side of the thumb, the palm, and back of the hand, lacerated. The crushed bones were removed at the nearest joints, and as much as possible saved, to form flaps, which were brought over the wound by adhesive straps, and water dressing applied. For some time she went on well, but on the night of the 6th of March, she was seized with irregular convulsions, for which an anodyne antimonial draught was ordered. On the 7th she was found unable to speak, and the jaw firmly locked, but she seemed sensible, and pointed to the region of the diaphragm: pulse regular, but weak. Six grains of calomel, followed by strong cathartic enemata, where ordered; the head shaved, and bathed with vinegar and water, and a blister applied to the scrobiculus cordis. At night a draught of Tinct. of Valerian, and Mur. of Morphiæ, was ordered. Next day the trismus continued, with occasional slight relaxations; the eyes were fixed and turned up, and the chest raised at times from the bed by a degree of opisthotonos. These symptoms continued, with slight remissions, for three or four days, during which time leeches and a blister were applied to the head. Large doses of Tinct. Opii administered; strong purgatives of calomel and jalap, followed by enemata, given; and the hand regularly poulticed. On the 10th, the tetanic symptoms had almost entirely subsided, and the hand looked well, but she complained much of her head. It was again blistered, and pills of Aloes, with Assafœtida given three times a day. From this time she improved regularly, and the hand continued to heal favourably till the 23d, when she was again attacked with trismus, and other tetanic symptoms. The head was again shaved, twelve leeches applied, followed by a blister, and a large dose of calomel, with cathartic enemata. This treatment was successful: the hand healed gradually, without any further alarming symptoms, and on the 7th of April she was dismissed cured.

This patient returned to the hospital on 24th of June, on account of severe epileptic attacks. She had continued well for eight or ten weeks after she was dismissed, but about a fortnight ago suddenly fell down in a fit; and these attacks gradually became more and more frequent. They now recur five or six times a day, and last about five minutes, after which she lies a considerable time in a state of stupor. As she was of a full habit, she was immediately cupped from the nape of the neck to the the extent of ten or twelve ounces.

℞ Calomel, gr. v.; Pulv. Jalapæ ℈i. M. Fiat pulvis, sint. vi. tales; Capiat unum indies.

26th.—No change. The fits are so severe that it has been found necessary to put her in the strait-waistcoat, to prevent her from injuring her head and arms against the walls and frame of the bed; and it requires two strong persons to keep her in bed. The head was shaved, and twelve leeches applied, and the purgatives continued. She also had occasionally a draught of Mur. of Morphiæ with Tinct. of Valerian.

20th.—Fits continue unabated.—Adhibeantur vesicatorium amplum Capiti raso.

July 4th.—There has been a slight improvement for the last two days. She is now taking the Assafœtida pills thrice a day.

6th.—Epileptic fits have returned with severity.

℞ Nitrat. Argenti. gr. iv. Micæ panis. q. s. M. Fiant pilulæ xii. Capiat unam bis die.

16th.—The nitrate of silver has been continued regularly, the dose being increased to half a grain a day, without any marked improvement.

Repetatur Vesicat. Capiti. Continuentur pilulæ Nitrat. Argenti, et Pulv. Jalapæ c. Calomel pro re natâ.

22.—As she still complained much of her head, it was determined to carry depletion still farther. Accordingly she was placed upright, and twenty-two ounces of blood taken from the arm, which produced slight faintness. Continuentur cætera.

30th.—Little improvement. It has been observed for the last few days, that, when the fits are slight, they are confined chiefly to the arm that was injured. On touching the stumps of the fingers smartly, the arm is convulsively withdrawn; and, when this is done while she lies in a state of stupor, violent convulsions of the arm are produced. She also says, when questioned, that she often feels a sensation proceeding from the injured hand, up the arm to her head, before the commencement of the fits.

August 10th.—Since last report the fits have been very severe, often confined to the arm, but always one or two general fits of great violence daily. As it seemed highly probable that the disease was occasioned by an affection of the extremities of the nerves in the injured hand, it was proposed in consultation either to amputate the hand, or cut down upon and remove a part of the nervous trunks leading to it. The first was preferred; first, because the hand was of little use, the remaining thumb being contracted and immoveable; and secondly, because, as the ends of all the metacarpal nerves seemed affected, it would be necessary to cut down upon the ulnar, as well as the median nerve, which would have rendered the operation as severe, and more tedious than amputation.

12th.—The amputation was performed today, near the middle of the forearm, by the double flap formed by transfixion. This mode was preferred, as being the most rapid, and allowing less time for the occurrence of the convulsions.

On tracing the nerves of the injured hand by dissection, the digital branches of the median nerve, and the branch of the ulnar, which supplies the ring finger, were found enlarged to four or five times their usual size, for one-third of an inch from their termination, and their extremities bulbous, and firmly imbedded in the hard cicatrix. They were also of a slight rose colour near their extremities, and firmer than usual. The flaps adhered by the first intention, and the cicatrization was almost complete on the 30th of August. The patient never had the slightest appearance of epilepsy after the operation, and was dismissed cured on the 8th of September.—*Aberdeen Infirmary Reports*, *in Lon. Med. Gaz.*

---

*Inhalation of Iodine and Conium in Tubercular Phthisis.* By CHARLES SCUDAMORE, M. D. —In my former reports, I had too much occasion to express my regret at the supineness of the profession at large in regard to inhalation; but I have now the satisfaction of stating, that I have received numerous communications from different parts of the kingdom, and some from abroad, giving me full and gratifying assurance of the great value of the remedy in question; of the sensible relief which it almost invariably affords; and of not unfrequent success in the removal of existing disease. Yet I am well aware that a great part of the profession have not thought proper to pay any attention to this new method of treatment; and some, I must say, are so illiberal as to condemn it in theory, never having tried it, choosing to assert that the vapour of iodine must be an irritant to the lungs, and, *ergo*, whatever irritates the mucous membrane must be injurious, and tend to aggravate the tubercular disease. All this is merely gratuitous assertion, and is directly contrary to fact. I am well convinced that the more this remedy is adopted, and carefully studied and understood, the more will it be approved. Let it not, however, be imagined, that I claim for it, boastfully, the power of curing the tubercular disease of the lungs in its worst forms; or that I allow myself to speak of tubercular phthisis as curable in a general sense; which might serve to imply that it is not the dangerous and commonly fatal disease which it has always been considered to be. My zeal for the remedy has never carried me to this imprudent length; but I may, on the other hand, be excused if I do not join in the despondency of those who almost shrink from contending with the disease, and who send away the unfortunate patient, in any stage of the disease except quite the last, to another climate. I hold this to be an exceedingly wise measure in in certain cases of the threatening of consumption, especially in young persons, whose constitution is not yet fully developed; but I also strongly condemn it, when serious disease has become established, requiring for its treatment the nicest means of art, and not a mere contentment with change of air, and climate, and attention to diet; advantages which are wholly inadequate to the cure, and too often insufficient even for the suspension of the disease. There are two conditions in which it is advisable that the invalid should pass the winter and early part of the spring in a favourable climate—the one already named, of the threatening of consumption at the early stage when the constitution is hovering on a doubtful balance; and the other for the purpose of counteracting relapse when a recovery from danger has been effected.

I am at a loss to know on what reasonable ground objections can be offered to the inhaling treatment in cases of consumption, in which the usual failure of all ordinary means is universally admitted. Besides that, the practitioner may use, in conjunction with it, any other measures to which he is attached: nor do I confine myself of the use of the single remedy—quite the contrary. I estimate very highly the advantages of counter-irritation; the modes of which are to be varied according to the case, and the choice is to be made between seton, issue, blisters, the solid or fluid, irritant liniments of different kinds, and rubefacients. As a general principle of practice, I advocate the use of tonics, which are various in their kind, and are to be chosen with reference to the particular case and constitution. Any accidental complication of other disease with that of the lungs is to be well considered; as disorder of the liver, and a morbid state of the mucous membrane of the intestinal canal, requiring a combined and alterative treatment. The administration of sedatives at night, when required to assist the sleep, allay cough, and prevent the wearing effects of restlessness and watchfulness, is very important. I always desire that the diet should be more than usually supporting, having due regard to the peculiar circumstances of the individual case and constitution.

Let it not, therefore, be said, that I have adopted the inhaling treatment on narrow grounds, and upon an empirical principle. When the idea of the remedy first* occurred to me, I reasoned on the probability that so active an agent as iodine, brought into immediate contact with the seat of disease, might be more effective in its influence than any other ordinary treatment; and I rejoice to say, that to a very great extent my most sanguine hopes and expectations have been fulfilled.

It will, perhaps, be said, that in the successful treatment of a case of consumption, so many other useful means being employed besides inhalation, it would be unjust to allow much credit to the latter remedy. But I assert that before I thought of it, and had recourse to it, I had, in very numerous cases, year after year, made use of of all other known means of treatment in vain,

* Ten years have elapsed since I published the first edition of my Treatise on Inhalations, and I made trials of the method for a long period before I published the results. About the time of my publication, Dr. Murray, of Belfast, made known the good effects of breathing iodine vapour diffused through the apartment; and, subsequently, Dr. Corrigan, of Dublin, has given his results from a similar mode of conveying iodine vapour to the lungs, using, however, a different method from Sir James Murray in procuring it. Either plan is altogether different from mine, of *direct* inhalation by means of a glass inhaler, so that the exact dose is as accurately administered as in the use of any internal medicine. Besides which, I claim credit for the superiority of the preparation of iodine which I employ, or rather its combination; and for the important modification of its action on the mucous membrane, which is derived from the addition to the iodine mixture of a saturated preparation of conium. I sometimes add ipecacuanha. In spasmodic asthma, I use other volatile medicines alternately with the iodine and conium.

sharing the common lot of failure and success; and I beheld consumptive disease as one beyond the reach of art. The power of iodine inhalation in controlling immediately the hectic fever, is no less remarkable than satisfactory.

In the volume of this Journal for 1834, I related a case which had been treated by Dr. Skrimshire, of Peterborough, and who, when he did me the favour to recommend the patient to my care, gave the following statement:—"This gentlemen is the subject of rapid tubercular phthisis. During the few weeks that he has been under my care, we have had recourse to occasional leeching, tartar emetic ointment and blisters, to acetic acid, to digitalis, hyoscyamus, &c. I have not, however, at any time, reduced the rapidity of the pulse, or the urgency of the cough for more than a day or two; the wasting has been progressive and rapid, and the expectoration, though never profuse, has, for the last three or four weeks, been puriform."

Nothing could be more satisfactory than the effect of inhalation of iodine and conium in this case. The hectic fever was immediately checked; the pulse, in a short time, became moderate, and every unfavorable symptom gradually gave way, so that the patient perfectly recovered, and he remains well up to the present day; and this is a happy and important point, which I would dwell upon with the highest gratification. The recoveries which I have at different times related, having given the details of the cases, have not been for merely a short period—a delusive convalescence, to end only in the bitterness of disappointment in fatal relapse, but lasting restoration to health and enjoyment. It is equally true, that I have often been consulted in the last stages of this sad disease, or, in some examples of its most inveterate nature, at an earlier period; and in either of which difficulties, if I had sought only the care of my own reputation, I would not have used inhalation, being certain of eventual failure; but the superior consideration of humanity always led me to employ the remedy for the sake of palliating suffering. A gentleman thus circumstanced with hopeless disease, declared that, although he was too well convinced he could not possibly recover, he wanted language to express his gratitude for the exceeding comfort and relief which he received from inhalation. It surely ought not to be stated as the reproach of any method of treatment, that it will not always succeed. In diseases of other organs of the body, as the stomach, the liver, or kidneys, or other parts, human skill will sometimes fail. How much greater is the difficulty of contending with disorganization of the lungs?

Yet, even in some very aggravated cases of tubercular excavation, I have been agreeably surprised by the recovery of the patient. In the case which I reported in the "Medical Gazette," vols. for 1840, the prognosis at first could only be unfavorable. Mr. King, of Portland-terrace, Regent's-park, attended the patient with me. I extract from his written statement the following particulars of the case: "The left lung gave strong puerperal resonance; the right lung was pectoriloquous, from the root to the mamma; there was much gurgling, and percussion was dull over the greater part of the right side; it was also much smaller than the left, and hollow at the clavicle. The body was much reduced; he had profuse hectic sweats, and the expectoration was copious, puriform, and very offensive; the pulse rapid. His debility was so great, that, to use his own words, he felt to be dying from day to day. The night perspirations were most profuse, and he was often sleepless. On the first inhalation, he expressed himself very sensibly relieved; afterwards his breathing was never oppressed in going up stairs, as it was before using the inhalation; and with a little more interval, he was able to walk two miles without fatigue."

I have lately seen this patient, and have the satisfaction of finding that there is every evidence of the cavity being almost healed, and the restored condition of the patient; his improved complexion, formerly so swarthy, from the imperfect function of the lungs; his increased bulk and accession of good strength, with altered spirits; and his former despair changed to confidence of attaining health, formed a picture at once most pleasing and satisfactory.

In the last winter I attended a gentleman, with Mr. Carter, of Dorset street, who labored under the most alarming symptoms from chronic bronchitis, and whose appearance wore the strong stamp of the last stage of tubercular phthisis. He was emaciated, and reduced to great debility; the pulse rapid; respiration labored and quick; cough extremely harassing, with foul and profuse expectoration, frequently also colored with blood; perspiration excessive; nights sleepless, and the apprehension of death. I persuaded myself that no ulceration had taken place, and I ventured to hope for success. Mr. Carter can bear his full testimony to the admirable effects produced by the inhalation of iodine and conium. The patient perfectly recovered.

Recently I have had a young gentleman under my care, recommended to me by Mr. Norton, of Dorset street, whose appearance was strongly consumptive. He was thin, pale, and much debilitated; night perspirations excessive; little sleep during three weeks, and the cough almost incessant day and night, with sputum of a suspicious character; oppression of the chest, with the breathing much hurried on slight exertion; loss of appetite and spirits; the pulse frequent and weak; the animal heat 99° to 100°. Respiration was scarcely audible over any part of the left side, and on percussion there was general dullness. The evidence of great tubercular obstruction was very clear; the signs on the right side were favorable. Already the happiest results from inhalation and other treatment have been produced. The cough became immediately relieved: appetite, sleep, and spirits have returned. At my first visit he was fatigued and distressed by only slight exertion; and now he can, without difficulty, walk three or four miles. In a word, the patient expresses himself to be getting well. I confidently expect his recovery.

With all the experience, therefore, which I have now enjoyed of the *methodus medendi* in consumption and chronic bronchitis, I trust I may be allowed to express myself in terms of that warm confidence and satisfaction, which a few trials and a shorter experience would not have warranted; and I will repeat the observations which I offered in my preface to the second edition of my Treatise on Inhalation.

"With my recorded conviction that the use of iodine by inhalation is productive of results highly and permanently beneficial; and that the constitution which prohibits its continuance is peculiar, and a rare exception to ordinary experience; I must express my earnest hope that the subject may receive from the profession at large such a full and dispassionate attention as is suited to its importance. It is not on selfish grounds that I advocate the practice. What concerns my reputation or advantage is personal and transient, and of little moment: what relates to science and to the interests of mankind, is for all ages, and of inestimable importance."

Deeply impressed, therefore, with the good results which my long experience had afforded me, I became desirous of contributing my exertions towards the establishment of an institution for the relief of the poor, afflicted with the diseases of the chest; that class of the community whose sufferings in illness are so bitterly aggravated by the stings of poverty, and who cannot command, like those more gifted by fortune, the advantages of good attendance and expensive medicines. I am happy to add, that this institution has taken root under the title of the *West London Dispensary for the Diseases of the Chest*, situate in Wells-street, Oxford-street.* During the last year great relief has been afforded to upwards of two hundred sufferers from consumption, bronchitis, and other affections of the air-passages, and from complaints of the heart; and many important cures have been effected, Very abundant further evidence has been obtained of the remarkable curative influence of inhalation; and to this mode of treatment, it is only justice to say that Mr. Carter, the acting surgeon of the institution, has paid the utmost attention, and with very great sucess.

It is always pleasing and satisfactory to me to quote the experience and the testimony of others, in regard to inhalation; not only as rendering a flattering support to my own views and opinions; but as affording an additional basis for the judgment of the profession at large. Up to a certain point scepticism is justifiable, and serves an useful purpose; but beyond that it sinks into unworthy and injurious prejudice.

The great experience of Sir Charles Scudamore requires us to pay more than a mere passing respect to his assertions. It is true that the benefits of this mode of treatment are limited, but still they are real, and therefore we are from time to time agreeably surprised at the unexpected results.

---

*Hypertrophy of the Abdominal Parietes.*—Margaret Adam, æt. 33, of a lax and rather thin habit of body, was admitted on the 6th of December, 1839, labouring under the following symptoms:—Belly is swollen, somewhat tense, tympanitic at its upper, and indistinctly fluctuating at its lower region. Complains of some weakness in back, but not of pain; pulse 86; tongue white; appetite very good; bowels slow; skin cool. Swelling commenced four months ago, shortly after childbirth, and was since continued; uterus normal. She was at this time treated with purgatives, diuretics, and mercury to salivation, and left the house with the swelling greatly diminished on the 1st of July, 1840. She returned on the 17th February, with similar symptoms. Belly much swollen, soft, and tympanitic, but no fluctuation can be felt: complains of occasional dyspnœa; appetite and general health good: bowels slow; swelling began about fourteen days ago; urine rather scanty, and deposits some flocculi on being heated.

Remarks.—This case is worthy of some observation, in as far as the diagnosis of abdominal diseases is concerned, for there was an apparent obscurity about the symptoms on the first examination, although it does not appear to be a disease attended with any particular, at least immediate danger. The abdomen, when percussed, has a peculiar doughy or fatty feel; at the same time it is accompanied with a low or concealed tympanitic sound. It may be characterized as a tympanitic sound passing through a denser medium than the abdominal parietes of normal thicknes; such as occurs in very fat individuals. This patient, however, is of rather a thin habit of body, so that a na-

* It is fully intended, as it is most earnestly desired, that this institution should grow into an infirmary or hospital for the reception of patients; the building being so constructed as to allow of the best system of ventilation in unison with regulated temperature; so important a desideratum in the treatment of pulmonary disease. The committee of management will wait only for the necessary funds to enable them to carry out this great object into full effect.

tural accumulation of fat in that situation cannot enter into the calculation; and she has no anasarca or fluctuation in the abdomen, while the integuments covering this latter cavity do not pit, in the slightest degree, by pressure; dropsical effusion must, therefore, also be excluded. On examining the abdomen with the stethoscope, the intestinal murmur was heard pretty distinctly, but deep-seated, or not in such close proximity to the ear as in a normal state of the abdomen, and, on pinching up a fold of the integuments from the abdominal muscles, it was found to be thickened to the extent of about two inches and a half; whereas, in most persons who are more than usually fat, the integument pinched up in this manner will not measure more than half an inch, and frequently less. What, then, is the disease of this patient since it is not ascites, anasarca, or a natural accumulation of fatty matter? It seems to depend upon two things, viz., first, the tympanitic swelling of the intestines, which does not exist to any great extent; and, second, the thickening of the integuments and cellular texture covering the abdominal muscles. Since her last admission she has been treated, in some respects, as formerly, with purgatives of the P. Jalap C. every second night, diuretic powders, and a blister to the abdomen, the latter being employed for the purpose of exciting absorption. She was dismissed on the 10th of April, with the abdomen very much reduced in size, the tympanitic swelling being altogether gone; but the integuments were still much thicker than natural.—*From a Clinical Lecture by Dr. Davidson, of Glasgow.*

---

*On the Structure and uses of the Lingualis Muscle, and its relations to those of the Panniculus Carnosus.* By James Mercer, M. D. F. R. C. S. E. Lecturer on Anatomy and Physiology, Edinburgh.—In man, and some others in the higher classes of animals, the tongue seems to serve in the function of taste alone; and on investigating the structure and conditions of its upper surface in these animals, this function can alone be assigned to it.

In taking a review, however, of its uses, in the various classes of the vertebræ, we cannot be justified in considering it as only an organ of taste in all of these. Even in some of the highest classes—the ruminantia and the felinæ—it principally serves for the prehension and taking in of their food; and it is at least very doubtful whether it possesses the sense of taste in several others: although, on the contrary, we would not be warranted in denying the existence of this sense in these animals, nor even in such as are entirely destitute of the organ, as the function can be performed by other parts; it not being the effect of a special and limited organ, but a property of the mucous membrane lining the whole of the cavity of the fauces.

It has been long known, that in most of the herbivorous mammalia, particularly the ruminantia, independent of the epithelium, the dermo-mucous membrane covering the upper surface of the tongue is of very firm and dense texture, and that it forms, as it were, appendages to itself—what the hairs, bristles, and quills, are to the surface of the body; numerous strong pointed papillæ, imbricated on each other, in a direction from the tip to the base of the organ.

In many of the felinæ, the marsupiatæ, and the bat tribe, the same peculiarity of structure takes place.

In several of the mammalia, the organ is not only used in the turning of the food from side to side, and for aiding in the primary part of the act of deglutition, but it also very materially, nay, in some very essentially, aids in the collecting of the food; whilst it at the same time serves the purpose of a comb or rasp for the animal cleaning itself. The ox, for instance, with his free tongue, rolls the herbage of the meadow into a kind of tuft, before he tears it through with the lower incisors and palate; and he also rasps and cleanse his own coat or that of his companion with its rough and prickly surface. In the dog and cat it is the sole agent, by means of which the fluids are conveyed to the mouth, by that action of the tongue called "lapping," and, in short, in every animal using the organ in a similar manner we have the papillæ on its upper surface correspondingly arranged and developed.

In all these animals also the muscular lamellæ of the lingualis are well developed, and very strong, immediately beneath the dense investing membrane of the tongue; and taking this fact, with the condition of the papillæ on its upper surface, with their evident mode of production and uses, it is curious that no other function should have been assigned to these muscles but that of "shortening the tongue and depressing its point,"—actions which we shall endeavour to show are only secondary, and the effect of another.

In the following remarks, therefore, we shall endeavour to point out more specifically what the real uses of these muscles may be; not only as these can be deduced from their anatomical arrangement and distribution, but also from analogical reasoning, in regard to their situation immediately beneath the epithelium and dermo-mucous covering of the tongue; serving to these structures, and their appendages, as we conceive, what the panniculus carnosus does to that of the cutaneous textures on the greater part of the surface of the body, viz: the erection or setting on end of these appendages, the papillæ.

The upper surface of the tongue in ruminating animals, and in the dog tribe, is covered over by a very dense and almost cartilaginous membrane, extending from the epiglottis forwards to the tip, and over its sides, where it becomes much changed in its appearance and structure, and takes on the nature of true mucous membrane.

Along the median line it is much thicker, and more dense, than at any other part, and appears in the dog, in the form of a distinct and rounded cord, commonly called the "worm of the tongue." This, by Caldani, was called the "linea albescens."*

Posteriorly this membrane is intimately attached to that which extends between the base of the tongue generally, the epiglottis and the os hyoides, and, in structure, appears evidently to be similar to the yellow elastic tissue.

On making an oblique section of the entire thickness of this membrane, it appears to differ somewhat in its two anterior thirds from that which is seen in the posterior; and hence it has been looked on by Gerdy, as warranting two distinct terms—the "*membrane folliculaire ou muqueuse linguale*," and the "*tissu folliculaire linguale.*"

The first of these, says he, " S'observe dans les deux tiers antérieurs de la membrane linguale, est grise, trés-dense, fort résistante, cartilaginiforme, et comme si elle était tapissée par un lame cartilagineuse."

The second, the "'tissue lingual folliculaire,' est placè sous la muqueuse, trés-mince de la partie supérieure de la base de la langue. Il occupe le tiers postérieur de la surface supérieure de celle-ci, environ tout l'espace qui est au-delà des papilles lenticulaires et coniques jusqu'à l'épiglotte, et on s'ouvrent les follicules si sensibles. Il adhére aussi très-fortement à la folliculeuse qui le recouvre immédiatement."†

This difference, however, appears to me to be more apparent than real; for, after the most careful examination of their minute structure in the ox and dog, no appreciable difference could be detected, and the only cognizable differences, the thickness and density, appearing rather to be dependent on the comparative absence of the papillæ on its upper surface.

On the two anterior thirds, where these projections most abound, there the tissue is most dense and strong, and its sensibility less than in the posterior third, which is well known to be, next to the margins of the tongue, in the animals above specified, the situation where the sense of taste is most acute over the surface of the organ. This opinion is still more strengthened by a comparative examination of this investing membrane, with that which is found along and under the margins of the tongue. There the structure seems to be in every respect similar to the "tissu folliculaire."‡

Projecting from the upper surface of the whole of this investing membrane, we have a great number of papillæ, varying much in size, form, and arrangement. They are largest and most prominent in the middle and anterior thirds, particularly along the centre of each section, but more scattered along the margins. In their directions, when viewed laterally, and the tongue of the animal is kept in the bottom of the mouth, they appear to lie flat along the surface, but imbricated on each other from before backwards; their apices looking towards the epiglottis.

No sooner, however, is any sapid body, or any other stimulus, applied to the surface, than they are immediately elevated. When thus seen in the tongue of the dog or cat, they appear arranged in comparatively regular and transverse lines, the apices then being lifted up, and their posterior surfaces rendered concave, so that we have now a series of small channels, well adapted for containing and supporting fluids, placed in them, and thereby transporting them into the back of the mouth, during the pendent position in which the head of the animal is then placed.

In their structure, they are nearly similar to that of the investing membrane, and their adherent surfaces are intimately attached to the anterior extremities of the fibres of the superficial lingual muscles.

Immediately beneath this investing membrane, we find the irregular bands of muscular fibres, to which the term *lingualis* has been applied. These bands have been arranged by Gerdy into four sets—"the superficial and deep, the vertical and tranverse lingual muscles;* but it is only the first of these which is the most conspicuous, and can alone act on the surface of the tongue and its papillæ. This muscle consists of two distinct lamellæ of fibres, extending from the base to the tip of the organ, and separated from each other by the linea albescens. In their general outline and configuration, they are slightly parabolic; their concavities looking towards each other along the median raphè, and their convexities to the margins of the tongue.

Posteriorly they arise by means of the glosso-epiglottic elastic tissue from the upper surface of the os hyoides, and from thence proceed forwards to different portions of the adherent surface of the investing membrane. The inner set of fibres are considerably the shortest; but they gradually become more lengthened as they are examined near the margins.

Their free or inserted extremities are undoubtedly into the whole of the lower surface of the investing membrane. This can be proved by two facts—first, by the roughened appearance of the dissection, after its most careful removal; this condition evidently depending on

*Icones Anatomicæ, Venet. 1804.

† Gerdy, Anat. et Physiolog. de Langue: Archives Gen. de Medecine, tom. vii. p. 363.

‡ See also Blandin, Archives Gen. de Med., 1823; also his These Inaugurale sur la structure de la langue du bœuf.

* Recherches d'Anatomie, etc. Paris, 1823, page 20.

the severing of muscular fibres from it in their transverse direction; and secondly, from our inability to trace, even with a glass, any of the muscular fibres forming it, curving downwards to the base of the organ,—the general tendency of all the muscles of the tongue, the intrinsic and extrinsic, being towards its upper surface and tip. Immediately beneath these layers of muscular fibres, we find another layer of comparatively loose cellular tissue, surrounding the free extremities of the different muscles of the body of the tongue. This layer is very vascular, being freely supplied from the terminal branches of the lingual arteries. The chief use of this layer, as we conceive, being to enable the superficial lingual muscles to be freely and easily moved from before backwards over the surface of the tongue, unconnected and unrestrained by the influence of the other muscles, from which they are thereby completely separated. Analogously considered, it is similar in situation and use to the adipose layer on the surface of the body, which separates the panniculus carnosus from those muscles which are in immediate connection with the trunk, and thereby confines its action to the common investing membrane and its appendages.

Having thus traced the fibres of the muscles, and seen that they are extended along the whole of the upper surface of the organ, and fixed posteriorly to the os hyoides, it will be easy to conceive that this latter must be the part whence it can become fixed before it can be called into action. Taking this, therefore, as its point of fixion whenever it contracts, either by the will of the animal, or by the presence of some stimulus, it becomes shortened in its length, and puckered, as it were, into transverse folds; and, it will also be observed, that, in consequence of the intimate attachment of the anterior fibres to the adherent surface of the investing membrane, this must also be shortened and wrinkled in a corresponding ratio.

No sooner, therefore, will this be effected, than its immediate free prolongations, the papillæ, will have their bases drawn backwards along with it, and with the natural consequence of lifting up and tilting forwards their apices; so that when the entire muscles are in a complete state of contraction, and the investing membrane shortened to its greatest possible condition, the whole of the papillæ on the dorsum of the tongue are raised up, and arranged, as already stated, in nearly regular undulating lines; serving thereby as so many tenter-hooks, for assisting in laying hold of the grass, in the herbivora; and forming so many hollow channels, or buckets, for lifting up, and containing safely—like the boxes of a circular chain-pump or dredging machine—the fluids, in such of the felinæ as take in this part of their food by the process of lapping.

The generally received opinion of the action of these muscles, viz., "of bending up the tip of the tongue," can only, therefore, be a secondary effect, following the perfect evolution of the papillæ; but we doubt much if the muscles ever act in that manner, there being a sufficient number of more powerful muscles to perform it.

*London Medical Gazette.*

